# Synergistic effect study on the co-delivery of paclitaxel and SiRNA targeting STMN1 based on MPDA nanoparticles in the therapy of ovarian cancer

**DOI:** 10.1186/s12951-025-03827-8

**Published:** 2025-12-09

**Authors:** Xueqian Qian, Yi Yuan, Yaning Zhang, Manlin Li, Xiaotong Wang, Weikai Li, Ling Wen, Guangxin Duan, Yangyang Han

**Affiliations:** 1 School of Life Science and Technology, Shandong Second Medical University, Weifang, 261053 Shandong P. R. China; 2 Center for Molecular Imaging and Nuclear Medicine, State Key Laboratory of Radiation Medicine and Protection, School for Radiological and Inter disciplinary Sciences (RAD-X), Soochow University, Collaborative Innovation Center of Radiological Medicine of Jiangsu Higher Education Institutions, Suzhou, 215123 China; 3 Department of Radiology, The Fourth Affiliated Hospital of Soochow University, Medical Centre of Soochow University, Suzhou, 215000 China

**Keywords:** MPDA, SiRNA, STMN1, PTX, Ovarian cancer

## Abstract

**Background:**

Ovarian cancer is one of the most prevalent gynecologic malignancies and ranks as a leading cause of death in female cancers. Chemotherapy remains the primary treatment modality, however, the development of chemoresistance poses a significant obstacle in ovarian cancer therapy, contributing to its high mortality rate.

**Results:**

In this paper, we report a multifunctional nanoparticle that enables laser-controlled release of co-loaded paclitaxel (PTX) and STMN1 siRNA for synergistic combination therapy. This nanoparticle can improve the microtubule stability by interfering the expression of STMN1, thereby increasing the sensitivity to PTX, blocking cells in the G2/M phase, and ultimately leading to cancer cell death. Additionally, it exhibits enhanced bioavailability, reduced systemic toxicity, and photothermal properties suitable for in vivo imaging.

**Conclusions:**

Our nanoparticle offers an innovative strategy for the concurrent therapy and monitoring of ovarian cancer, enhancing therapeutic efficacy and diagnostic precision.

**Graphical abstract:**

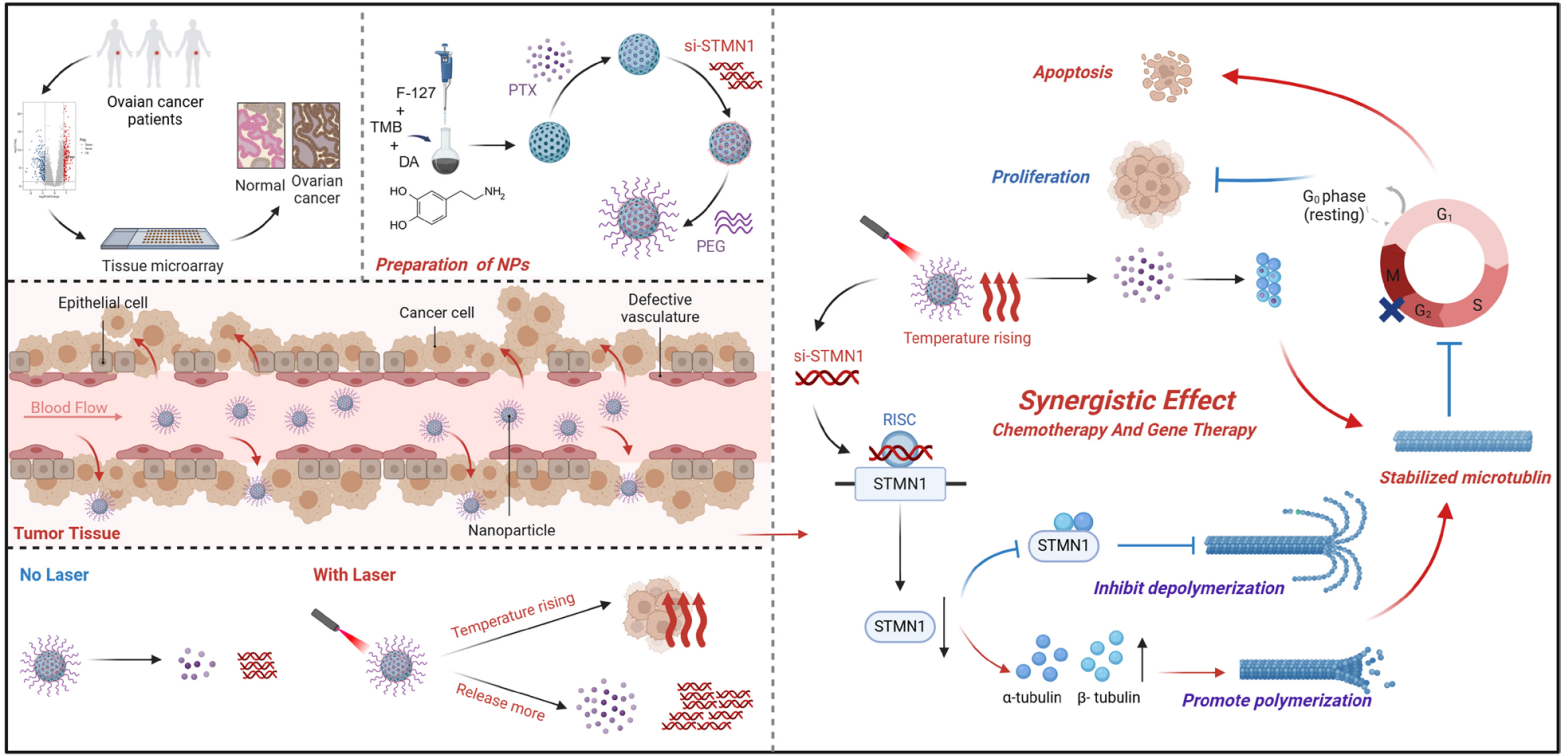

**Supplementary Information:**

The online version contains supplementary material available at 10.1186/s12951-025-03827-8.

## Background

Ovarian cancer is one of the most common gynecologic malignancies, ranking among the leading causes of death in female cancers, with a 5-year overall survival rate of approximately 40% [1, 2]. During the malignant progression of cancer, gene mutations or expression alterations frequently occur, contributing to chemoresistance [3, 4]. Moreover, drug resistance remains a major bottleneck in the treatment of ovarian cancer, resulting in a mortality rate of approximately 90% among patients with advanced disease [5–7]. Increasing evidence suggests that aberrant regulation of microtubule-associated proteins plays a critical role in this process [8, 9]. Among them, stathmin 1 (STMN1), a microtubule-regulating protein that promotes microtubule depolymerization by binding to α/β-tubulin heterodimers, has been reported to be highly expressed in multiple cancers and associated with poor prognosis [10, 11], including nasopharyngeal carcinoma (NPC) [12], non-small cell lung cancer (NSCLC) [13], breast cancer [14] and hepatocellular carcinoma (HCC) [15, 16], etc.

Moreover, STMN1 plays an essential role in various critical cellular processes, including cell cycle progression, differentiation, apoptosis, and cellular motility [17–19]. Notably, bioinformatics analysis and our ovarian tissue microarray data demonstrated a significant upregulation of STMN1 expression in ovarian cancer. However, the precise role and underlying mechanisms of STMN1 in the progression of ovarian cancer require further investigation.

Paclitaxel (PTX), a taxane-class chemotherapeutic agent, is currently utilized as a first-line drug in the clinical management of ovarian cancer [20]. By stabilizing microtubules and disrupting their normal dynamics, PTX induces mitotic arrest, ultimately resulting in cell death [21]. Several existing studies have suggested that resistance to PTX analogues may arise from altered interactions among microtubule proteins, including STMN1 in breast cancer [22, 23]. However, in ovarian cancer, this hypothesis has not yet been substantiated by research results.

To enhance the bioavailability of PTX, maximize cancer cell sensitivity to the drug, and preserve patient health, nanotechnology-based drug delivery systems co-delivering siRNA and PTX have emerged as a potentially pivotal therapeutic strategy. Therefore, we selected mesoporous polydopamine (MPDA) owing to its ability to accommodate biological macromolecules through its adjustable pore size and depth, enabling controlled or sustained release triggered by pH changes or second near-infrared (NIR) lasers irradiation [24]. Additionally, the intrinsic photothermal conversion property of MPDA enables it to convert light energy into heat energy under NIR laser irradiation, thereby enhancing thermal therapy.

In this study, we demonstrated STMN1 as a potential clinical therapeutic marker in ovarian cancer and successfully designed and constructed a PEG-coated nano delivery system based on MPDA, which co-loaded with PTX and siRNA (STMN1) for the combined therapy of ovarian cancer. PTX/si@MPDA-PEG exhibited a significantly improved therapeutic benefit by enhancing microtubule stability, thereby significantly increasing the chemosensitivity of ovarian cancer cells to paclitaxel and markedly reducing cell migration. Additionally, it induced pronounced cell cycle arrest at the G2/M phase. Collectively, these findings provide a novel and promising therapeutic approach for ovarian cancer.

## Results

### STMN1 serves as a promising diagnostic and prognostic biomarker in ovarian cancer

**Fig. 1:**
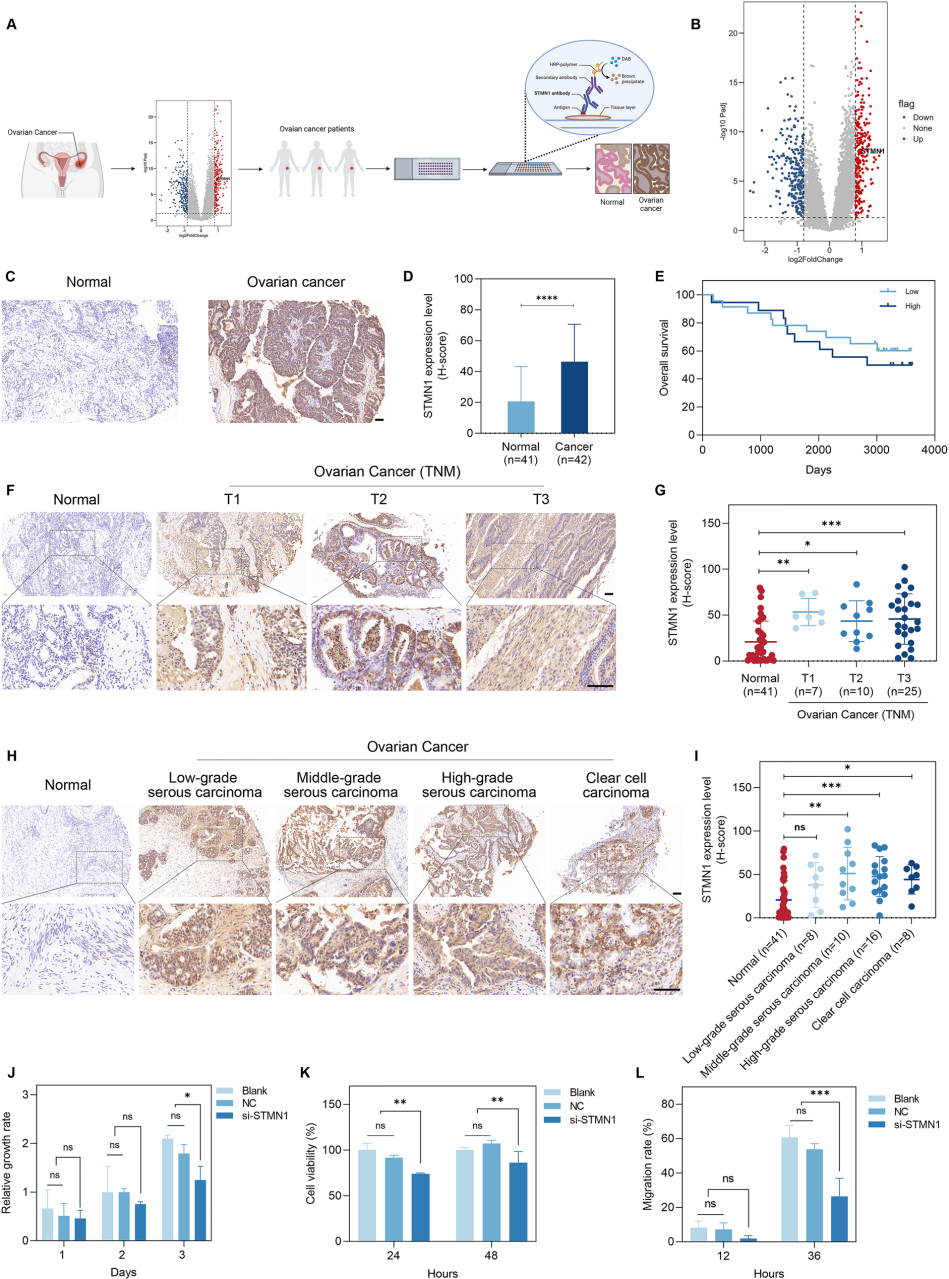
STMN1 functions as a potential therapeutic target and prognostic indicator in ovarian cancer. (**A**) A schematic illustration of gene expression analysis from clinical database and immunohistochemical staining of clinical samples. **(B**) Volcano plot representation of differential protein expression in tumor versus normal tissues. (**C**) Expression of STMN1 in ovarian cancer tissues and adjacent normal tissues. (**D**) Graph of the H-score analysis of STMN1 expression shown in panel C. (**E**) Survival analysis of ovarian cancer patients based on STMN1 expression levels. (**F**) Expression of STMN1 in adjacent normal tissues and ovarian cancer tissues from different pathological stages, and (**G**) corresponding statistical analysis. (**H**) Expression of STMN1 in adjacent normal tissues and ovarian cancer tissues from different subtypes, and (**I**) corresponding statistical analysis. (**J**) Relative growth rate of cells in different treatment groups after three days of culture. (**K**) Cell viability in different treatment groups at 24 and 48 h, as measured by the CCK-8 assay. (**L**) Analysis of wound healing in the wound healing assay in different groups. Scale bar: 50 μm. *p < 0.05, **p < 0.01, ***p < 0.001, ****p < 0.0001, n.s.: no significance

To elucidate the precise role of STMN1 in ovarian cancer, both bioinformatical analysis and tissue microarray analysis were conducted (Fig. 1). The results demonstrated that STMN1 expression was significantly higher in ovarian cancer tissues compared to normal ovarian tissues (Fig. 1A-D). Besides, in the Kaplan-Meier survival curve analysis, the high expression of STMN1 was associated with poorer overall survival rates in the entire cohort of ovarian cancer patients (Fig. 1E). Furthermore, STMN1 exhibited significantly elevated expression across various pathological stages and different subtypes of ovarian cancer (Fig. 1F-I).

To further investigate the role of STMN1 in ovarian cancer progression, STMN1 was silenced to examine its effects on the functionality and behavior of ovarian cancer cells in vitro. First, siRNAs targeting STMN1 were designed and synthesized, and the knockdown efficiency of the three siRNAs was confirmed. The results demonstrated that in the si-381 group, STMN1 protein expression was markedly reduced compared to the control group, achieving the highest knockdown efficiency (Figure S1, S2). We further validated these findings in another ovarian cancer cell line, SKOV3, and obtained consistent results (Figure S3, S4).

Next, cell proliferation assays, CCK-8, and scratch wound assays were conducted to examine the effects of STMN1 on ovarian cancer cell proliferation. The results demonstrated that, compared to the control group, the knockdown of STMN1 resulted in a significant reduction in both ovarian cancer cell proliferation and wound healing rate (Fig. 1J-L, Figure S5), further substantiating the critical role of STMN1 in ovarian cancer progression. Consistent findings were observed in the SKOV3 cell line (Figure S6-S8).

## Preparation and characterization of PTX/si@MPDA-PEG

**Fig. 2:**
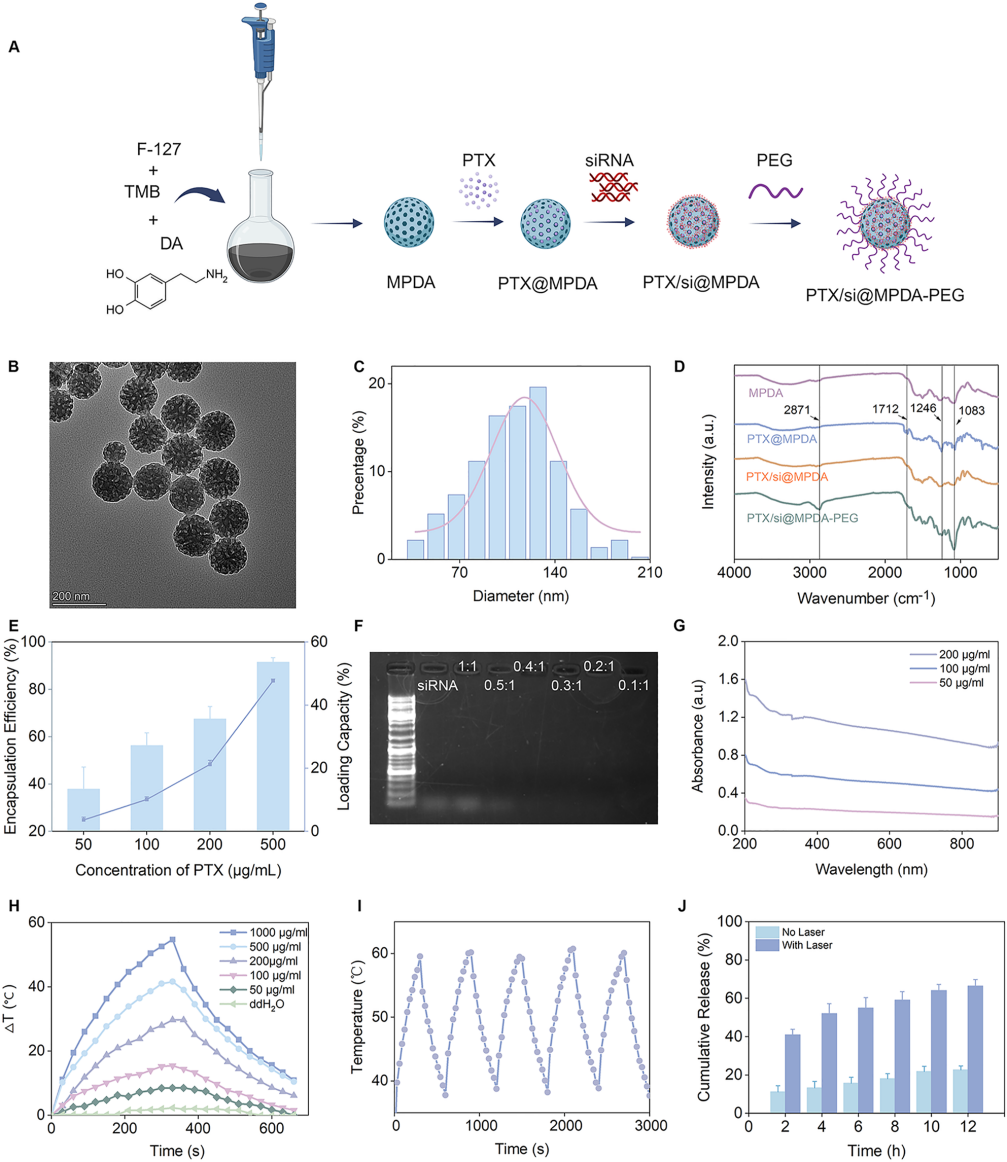
Preparation and Characterization of PTX/si@MPDA-PEG. (**A**) Synthetic schematic diagram of PTX/si@MPDA-PEG. (**B**) TEM images of MPDA. (**C**) Statistics of particle size. (**D**) FT-IR spectra of MPDA, PTX@MPDA, and PTX/si@MPDA, and PTX/si@MPDA-PEG nanoparticles. (**E**) The PTX loading capacity and encapsulation efficiency (**F**) Agarose gel analysis with different weight ratios of siRNA/MPDA. (**G**) UV-vis absorbance spectra of MPDA nanoparticles at different concentrations. (**H**) Photothermal curves of PTX/si@MPDA-PEG of different concentrations under 808 nm laser (2 W/cm^2^). **(I)** Five cycle heating curves of PTX/si@MPDA-PEG under 808 nm laser irradiation (2 W/cm^2^). (**J**) The PTX release performance of PTX/si@MPDA-PEG with or without irradiation (808 nm, 2 W/cm^2^, 5 min) at an interval of 2 hours

MPDA with a well-defined mesoporous structure was synthesized using an improved soft-template method [24] (Fig. 2A). Transmission electron microscopy (TEM, Fig. 2B) and scanning electron microscopy (SEM, Figure S9) images revealed that the MPDA nanoparticles were uniformly distributed. The majority of the nanoparticles exhibited a size of approximately 120 nm (Fig. 2C), featuring well-ordered pore structures. The hydrated particle size of MPDA was approximately 142 nm, while after loading PTX and siRNA onto MPDA, the average hydrated particle size increased to approximately 190 nm (Figure S10). The final zeta potential was − 4.3 mV (Figure S11), which can reduce non-specific adsorption during delivery, enhance nanoparticle stability in physiological media, and improve biocompatibility [25]. The polydispersity index (PDI) of the nanomaterials in various solution system remained relatively constant over a period of 72 h, indicating that the systems exhibited good stability (Figure S12).

Fourier transform infrared (FTIR) spectroscopy was used to characterize the synthesized nanoparticles. As shown in Fig. 2D, the MPDA spectrum exhibited a broad and intense absorption peak between 3000 and 3500 cm^− 1^, which can be attributed to the stretching vibration of intermolecular hydrogen bonds (O-H) or the aromatic secondary amine (N-H) stretching vibration. The absorption peaks at 1600 cm^− 1^ and 1511 cm^− 1^ correspond to the stretching and deformation vibrations of the benzene ring. An ester absorption peak at 1712 cm^− 1^ is the C = O stretching vibration of PTX molecules, while the characteristic absorption at 1246 cm^− 1^ corresponds to the C-O vibration, thereby confirming the successful incorporation of PTX into the mesoporous polydopamine structure [26]. In addition, a significant enhancement in the stretching vibrational peaks of the siRNA phosphoryl group in the range of 1080–1250 cm^− 1^ was observed, demonstrating successful loading of the siRNA. Furthermore, the appearance of a C-O-C stretching vibration peak at 1050–1150 cm^− 1^ further validated the successful encapsulation of PEG.

The N₂ adsorption-desorption isotherm exhibits a typical Type IV with a Type H4 hysteresis loop, confirming the mesoporous feature of MPDA (Figure S13). The Barrett-Joyner-Halenda (BJH) adsorption differential pore size distribution shows a dominant pore diameter of approximately 400 Å, along with minor peaks in the range of 100–300 Å, indicating that the pore sizes predominantly distributed within the mesoporous range of 100–400 Å (Figure S14). These results further support that the MPDA possesses a well-defined mesoporous structure. The uniform pore distribution and open pore channels offer suitable pore size compatibility and sufficient pore volume for the effective drug loading.

The loading capacity of MPDA for siRNA and PTX was further evaluated by varying the feeding ratios. The results indicated that as the feed ratio of PTX increased, the drug loading capacity gradually increased, reaching a maximum of 47.8%, with a corresponding encapsulation efficiency of 91.4% (Fig. 2E). The agarose gel electrophoresis results indicated that siRNA could be completely loaded when the w: w ratio was 0.4:1 (Fig. 2F). Besides, UV absorption spectroscopy results showed that MPDA exhibited continuous absorption between 200 and 900 nm without a distinct peak (Fig. 2G).

Accordingly, we investigated the temperature response of PTX/si@MPDA-PEG under 808 nm laser irradiation. The results revealed that the photothermal performance was concentration-dependent, with excellent photothermal stability (Fig. 2H, I). Specifically, a maximum temperature of 61 °C was achieved at a concentration of 200 µg/ml, and the visualization image was shown in Figure S15. Additionally, under multiple irradiations of the 808-nanometer laser, the cumulative PTX release reached approximately 66%, which is approximately three times higher than that at normal body temperature (37 °C) (Fig. 2J).

## PTX/si@MPDA-PEG exhibited significantly enhanced therapeutic potential of ovarian cancer in vitro

**Fig. 3:**
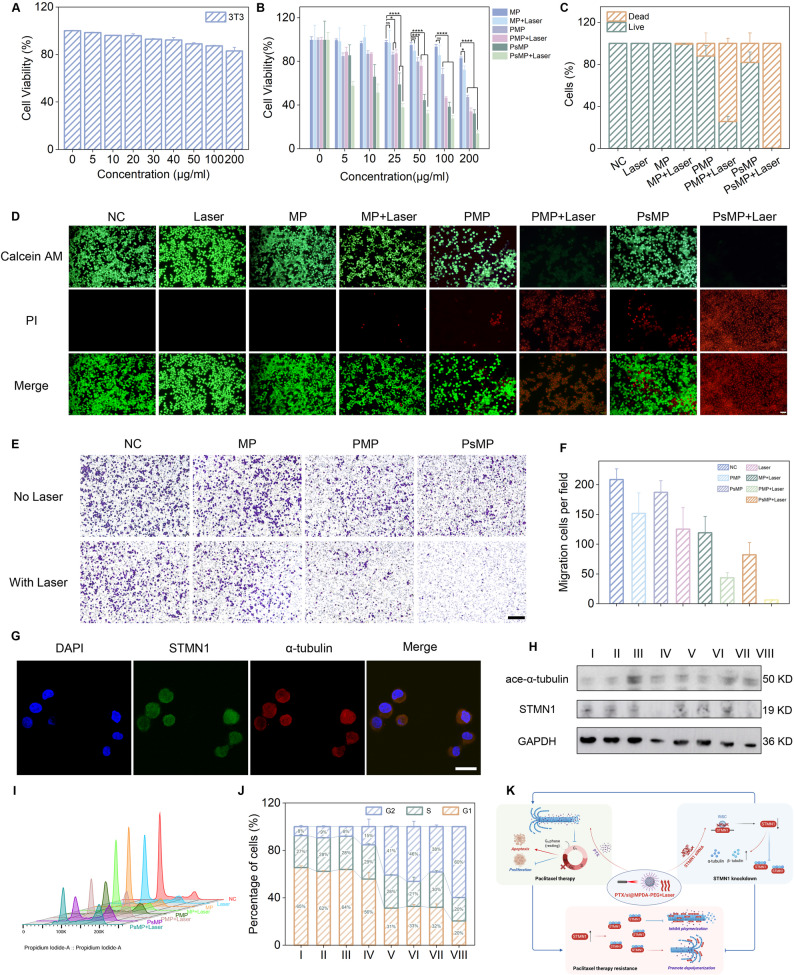
Anti-tumor effect of PTX/si@MPDA-PEG in vitro. (**A**) Cell viability of 3T3 cells incubated with MPDA-PEG nanoparticles for 24 h with different concentrations. (**B**) In vitro therapeutic benefit of different treatment groups at various concentrations. Data analysis (**C**) and CLSM images (**D**) of Calcein AM (green fluorescence, live cells) and Propidium iodide (PI, red fluorescence, dead cells) co-stained A2780 cells. Scale bar: 50 μm. (**E**) and (**F**) The Transwell assay to assess the impact of different treatments on the migratory ability of ovarian cancer cells after co-incubation with A2780 cells. Scale bar: 50 μm. (**G**) STMN1 colocalized with microtubules in A2780 cells. Scale bar: 25 μm. (**H**) Western blot grayscale images of STMN1 and ace-α-tubulin expression after different treatments. I: NC, II: MPDA-PEG (MP), III: PTX@MPDA-PEG (PMP), IV: PTX/si@MPDA-PEG (PsMP), V: Laser, VI: MPDA-PEG + Laser (MP + Laser), VII: PTX@MPDA-PEG + Laser (PMP + Laser), and VIII: PTX/si@MPDA-PEG + Laser (PsMP + Laser). (**I**) and (**J**) Cell cycle analysis of A2780 cells pretreated with different treatments. I: NC, II: Laser, III: MPDA-PEG (MP), IV: MPDA-PEG + Laser (MP + Laser), V: PTX@MPDA-PEG (PMP), VI: PTX@MPDA-PEG + Laser (PMP + Laser), VII: PTX/si@MPDA-PEG (PsMP), and VIII: PTX/si@MPDA-PEG + Laser (PsMP + Laser). (**K**) A schematic illustration of the synergistic mechanism for overcoming PTX-induced chemoresistance. *p < 0.05, **p < 0.01, ***p < 0.001, ****p < 0.0001, n.s.: no significance

A thorough safety assessment of MPDA-PEG was conducted as a priority before initiating in vitro therapeutic studies. Normal cell lines were co-incubated with varying concentrations of MPDA-PEG, and cytotoxicity was assessed using the CCK-8 assay. The results showed that MPDA-PEG maintained a high survival rate of about 83% at concentrations up to 200 µg/mL (Fig. 3A), suggesting that MPDA has excellent biocompatibility. Then, various groups of nanomaterials were co-incubated with A2780 cells to detect the anti-tumor activity, and as the result shown in Fig. 3B, the PTX/si@MPDA-PEG group exhibited the highest therapeutic benefit at all the concentrations, suggesting that STMN1 interference significantly enhanced the chemotherapeutic sensitivity of ovarian cancer cells to PTX (Fig. 3B). Furthermore, consistent results were observed using Calcein-AM/PI staining (Fig. 3C, D). Next, the Transwell assay was used to assess the invasive ability of the tumor cells, and in the PTX/si@MPDA-PEG + Laser group, almost no cells migrated from the upper chamber to the lower chamber, which showed the best migration inhibition effect (Fig. 3E, F).

Based on previous studies, STMN1 is a microtubule-associated protein that is predominantly localized in the cytoplasm. Therefore, we visualized the intracellular distribution of STMN1 using confocal microscopy (Fig. 3G). PTX-induced cytotoxicity primarily relies on microtubule stabilization, and enhanced sensitivity to PTX is likely attributed to increased microtubule stability [27]. Acetylated tubulin serves as a marker of microtubule stability [28]. Accordingly, we examined changes in the levels of acetylated tubulin. The results showed that, upon silencing STMN1, the intracellular levels of acetylated tubulin increased (Fig. 3H), further supporting the hypothesis that the enhanced therapeutic effect is attributable to increased sensitivity to PTX. Flow cytometry results demonstrated that the PTX/si@MPDA-PEG group exhibited the most pronounced G2/M phase arrest (Fig. 3I, J). Taken together, these results confirm that silencing of STMN1 promotes the therapeutic effects of PTX by enhancing the microtubule-stabilizing effect and inducing G2/M phase arrest (Fig. 3K).

## Excellent optoacoustic (OA) imaging performance and synergistic antitumor efficacy of PTX/si@MPDA-PEG in vivo

**Fig. 4:**
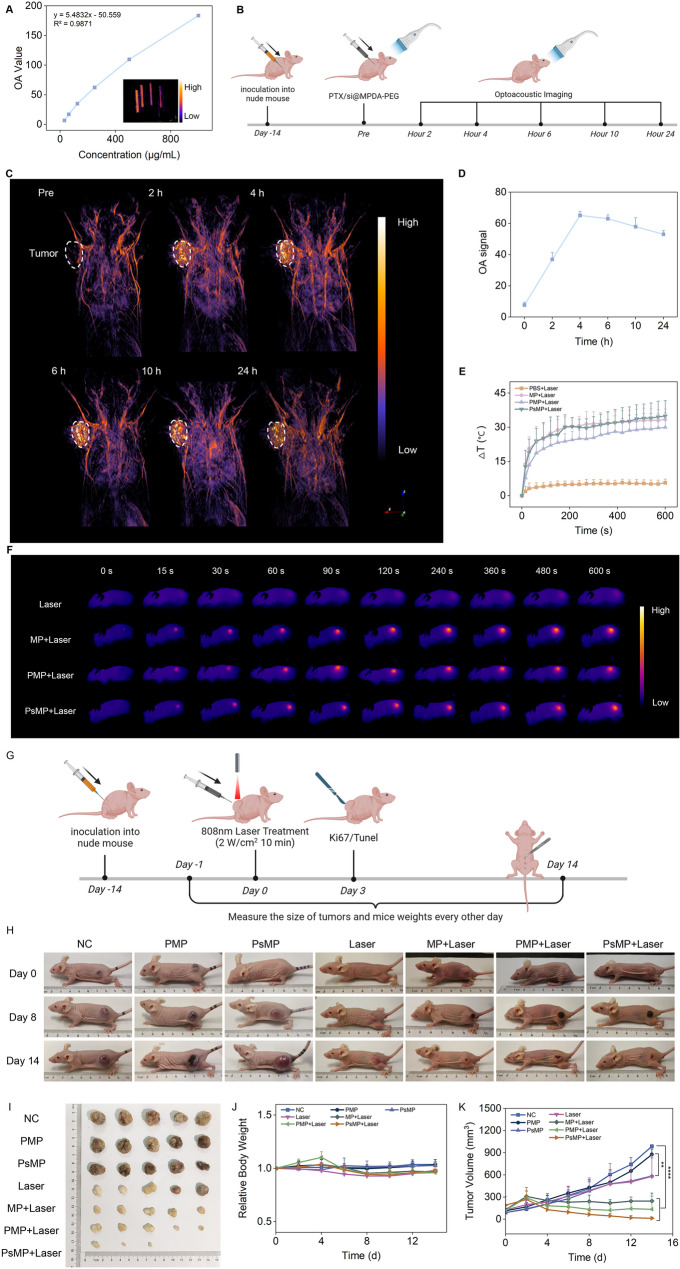
OA imaging performance and in vivo antitumor activities of PTX/si@MPDA-PEG. (**A**) OA signals of the indicated concentrations of PTX/si@MPDA-PEG nanoparticles in water in a thin tubing. (**B**) Schematic illustration of in vivo OA imaging of tumor-bearing nude mice. (**C**) Time-dependent PA images of tumor sites after intratumoral injection of PTX/si@MPDA-PEG nanoparticles in tumor-bearing nude mice. (**D**) OA signals in the tumor region after injected PTX/si@MPDA-PEG nanoparticles are recorded at different time points. Local temperature (**E**) and thermal images (**F**) of four groups of A2780 tumor-bearing nude mice. (**G**) Schematic illustration of in vivo treatment of tumor-bearing nude mice. (**H**) Photos of tumors dissected from each group on the 14th day after treatment. (**I**) Digital photos of A2780 tumor-bearing mice from each group on 0, 8, and 14 days. (**J**) Relative body weight and (**K**) tumor volume of A2780 tumor-bearing mice with different treatments. PBS (NC), PBS + Laser (Laser), MPDA-PEG + Laser (MP + Laser), PTX@MPDA-PEG (PMP), PTX@MPDA-PEG + Laser (PMP + Laser), PTX/si@MPDA-PEG (PsMP), and PTX/si@MPDA-PEG + Laser (PsMP + Laser). Data are presented as mean ± SD (*n* = 5). *p < 0.05, **p < 0.01, ***p < 0.001, ****p < 0.0001, n.s.: no significance

Another significant advantage of MPDA is its capability to be visualized through OA imaging. To evaluate the OA performance of the nanoparticle system in vitro, we conducted tests across various concentration gradients. The results demonstrated a strong linear relationship between OA signal and concentration, indicating suitability for OA imaging experiments (Fig. 4A). Following intratumoral injection of the nanoparticles, OA imaging was employed to monitor the accumulation of PTX/si@MPDA-PEG within the tumor (Fig. 4B). The data revealed that signal intensity reached its peak at 4 h post-injection and subsequently declined, yet remained detectable for over 24 h, showcasing excellent resolution (Fig. 4C, D). In contrast, after tail vein injection, the peak signal intensity at the tumor site was observed approximately 6 h post-injection, with sustained retention also noted (Figure S16, S17).

Based on the aforementioned findings, we monitored the temperature changes at the tumor site using a thermal imaging system following intratumoral injection and subsequent laser irradiation. The results demonstrated that after 10 min of 808 nm laser irradiation, the tumor temperature increased from 34 °C to approximately 68 °C. This temperature elevation not only facilitated the further release of PTX and STMN1-siRNA but also reached the optimal temperature required for effective tumor hyperthermia. (Fig. 4E, F).

The favorable controlled release effects and photothermal therapy performance of PTX/si@MPDA-PEG highlight its potential as an antitumor nanomaterial. To further evaluate its anti-tumor efficacy, A2780 tumor-bearing mice were randomly assigned to seven groups and subjected to different treatments: PBS (NC), PBS + Laser (Laser), MPDA-PEG + Laser (MP + Laser), PTX@MPDA-PEG (PMP), PTX@MPDA-PEG + Laser (PMP + Laser), PTX/si@MPDA-PEG (PsMP), and PTX/si@MPDA-PEG + Laser (PsMP + Laser). Following tumor implantation, drug administration, and irradiation treatment, the tumor volume and body weight of the mice were monitored every other day for 14 days (Fig. 4G). Notably, the tumor progression in nude mice injected with PTX/si@MPDA-PEG was significantly inhibited, which can be attributed to the suppression of STMN1 expression, thereby enhancing chemosensitivity to PTX. Furthermore, the combination of PTX/si@MPDA-PEG with laser therapy markedly amplified the tumor-killing effect on the 14th day, leading to the elimination of even larger tumors. This outcome provides compelling evidence of its potent synergistic anti-tumor efficacy (Fig. 4H-K).

## Evaluation of the tumor inhibitory effect of the combination therapy and safety of PTX/si@MPDA-PEG in vivo

**Fig. 5:**
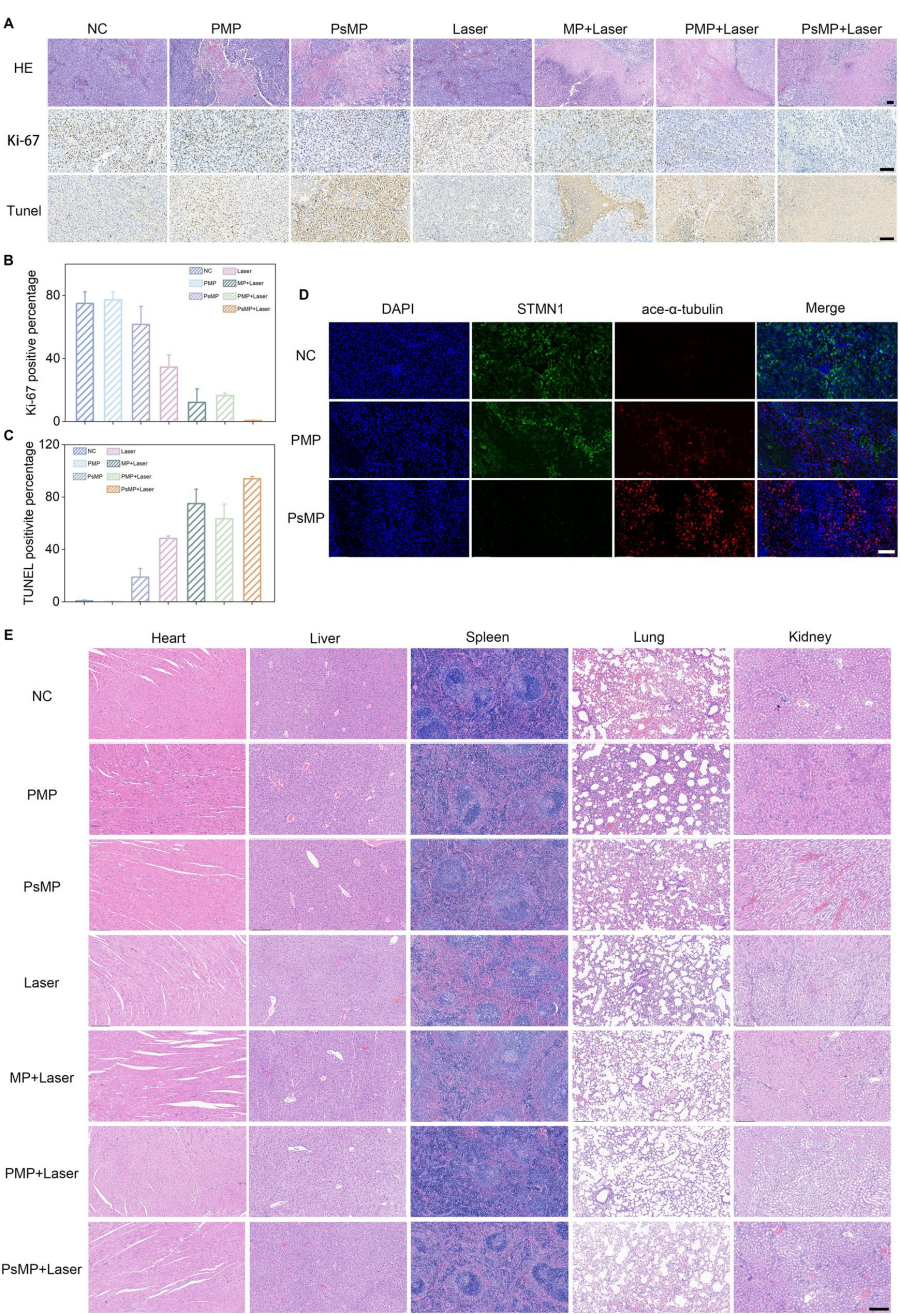
The impact of combined therapy on tumor regression and organ function *in vivo.* (**A**) H&E, Ki67, and TUNEL staining images of tumor tissues after different treatments. Scale bar: 100 μm. (**B**) Quantification of Ki67 and (**C**) TUNEL-positive cells expressed as the percentage of total cells. **(D**) The expression of STMN1 and ace-a-tubulin in different treatment groups was assessed. Scale bar: 100 μm. (**E**) H&E staining images of major organs (heart, liver, spleen, lung, and kidney) and tumor tissues dissected from each group on the 14th day after treatment. Scale bar: 200 μm. PBS (NC), PBS + Laser (Laser), MPDA-PEG + Laser (MP + Laser), PTX@MPDA-PEG (PMP), PTX@MPDA-PEG + Laser (PMP + Laser), PTX/si@MPDA-PEG (PsMP), and PTX/si@MPDA-PEG + Laser (PsMP + Laser)

After therapy, the tumor tissue in the PTX/si@MPDA-PEG + Laser group exhibited substantial damage, as evidenced by a decreased nuclear-to-cytoplasmic ratio in tumor cells, an increased TUNEL signal indicating elevated apoptosis rates, and reduced expression of the cell proliferation marker Ki-67 (Fig. 5A-C).

In addition, immunofluorescence staining of tumor tissue sections demonstrated that STMN1 interference led to a significant reduction in green fluorescence-labeled STMN1. Concurrently, the combination of PTX and STMN1-siRNA markedly enhanced red fluorescence labeling of acetylated microtubules. These observations further indicate increased expression levels of acetylated microtubules and improved microtubule stability (Fig. 5D). These findings are consistent with the results obtained from in vitro experiments.

Finally, the in vivo biocompatibility of the nanoparticle drugs was systematically evaluated. Histological examination via H&E staining of major organs (heart, liver, spleen, lungs, and kidneys) from mice in each treatment group revealed no significant pathological alterations (Fig. 5E). Additionally, blood samples collected from mice in each treatment group were analyzed for routine hematological parameters. The results indicated that there were no notable differences in red blood cell count (RBC), white blood cell count (WBC), hemoglobin levels (HGB), platelet count (PLT), and other relevant indices (Figure S18).

## Discussion

Stathmin (also known as p18), a cytoplasmic protein encoded by the STMN1 gene, is widely expressed and primarily localized in the cytoskeleton [29]. It is frequently overexpressed in various malignancies and has been shown to be closely associated with poor prognosis [16, 22]. Our findings in this study revealed that the expression of STMN1 was significantly higher in ovarian cancer tissues compared to normal tissues, which was associated with shorter overall survival (Fig. 1A-I, S1-S8). These results are consistent with previous studies in ovarian cancer [30]. However, the expression of STMN1 did not exhibit a consistent increase aligned with the progression of TNM stages (Fig. 1F, G). This inconsistency may be attributed to two factors: first, the microarrays of ovarian cancer used for IHC comprised various subtypes of ovarian cancer tissues, and STMN1 expression levels differed significantly among these subtypes (Figure H, I). Second, this variation might also stem from the limited number of tissues, including both normal and malignant tissues. Nonetheless, the precise role of STMN1 in ovarian cancer and its clinical implications warrant further elucidation.

PTX, a first-line chemotherapeutic agent for a variety of malignancies, including ovarian cancer, primarily exerts its anti-tumor effects by disrupting the dynamic balance of microtubules, causing cell cycle arrest at the G2/M phase, thereby preventing normal mitosis and subsequently triggering apoptosis, which ultimately results in tumor inhibition or eradication [31–34] However, approximately 90% of patients with advanced ovarian cancer develop chemoresistance, leading to a failure in benefiting from PTX treatment [35–37]. And this is mainly due to alterations in tubulin interactions, particularly changes in microtubule-protein interactions, such as those involving stathmin [20].

In this study, we developed a PEG-coated nano-delivery system, PTX/si@MPDA-PEG, which enables controlled release of PTX and siRNA under 808 nm laser irradiation (Fig. 2, S9-14). This system overcomes the barriers associated with the co-delivery of free PTX and siRNA. It not only addresses the poor water solubility and severe side effects of PTX, which have been partially mitigated by previous strategies but remain challenging due to its unique nonlinear pharmacokinetic properties [38], but also achieves an optimal balance between the efficacy and toxicity of PTX.

Furthermore, both in vitro and in vivo experiments confirmed the potent anti-tumor efficacy of PTX/si@MPDA-PEG. In vitro, PTX/si@MPDA-PEG (especially with laser) significantly inhibited ovarian cancer cell proliferation/migration, increased ace-α-tubulin (microtubule stability marker), and induced G2/M arrest (Fig. 3). And in vivo, it nearly eliminated tumors in nude mice with no major organ toxicity (Figs. 4 and 5, S18). We speculate that the laser intervention caused local heating of the tumor site, which disrupts the weak interaction between PTX and MPDA, allowing more PTX and siRNA to be released [39]. In addition, under high-temperature conditions, the enhanced molecular motion within the pores of MPDA may further accelerate the diffusion and release of PTX [40]. Meanwhile, the hyperthermia effect resulting from the local temperature increase cannot be ignored, which further ensures the effective elimination of tumor cells and jointly contributes to the significant anti-tumor effect. However, despite the advantages of controllable release characteristics, including low toxicity and high therapeutic efficacy, from the perspective of clinical translation, several concerns remain. These include potential cumulative toxicity of PEG-coated nanoparticles, such as their impact on liver and kidney function, as well as whether localized hyperthermia might cause deep tissue damage or immunosuppression. Such issues still require further verification through long-term repeated administration experiments [41].

The applicability of enhanced permeability and retention (EPR) effects serves as the cornerstone for the passive targeting mechanism of PTX/si@MPDA-PEG. The hydrated particle size of PTX/si@MPDA-PEG is 190 nm (Figure S10), which falls within the optimal range for EPR-mediated extravasation (100–200 nm) [42]. Additionally, the near-neutral zeta potential (−4.3 mV, Figure S11) minimizes nonspecific interactions with blood components, thereby reducing premature uptake by the reticuloendothelial system and enhancing contact with tumor vasculature. Moreover, the excellent stability of the nano-delivery system also ensures structural integrity throughout blood circulation (Figure S12).

In addition to therapeutic efficacy, the nanoparticles enabled dual monitoring through optoacoustic and thermal imaging, providing valuable information on drug release regulation and treatment monitoring [43]. OA imaging results demonstrate that the nanoparticle system effectively achieves passive targeting via EPR effects, while prolonging blood circulation time and maintaining sustained tumor accumulation (Fig. 4A-D, S16-S17). Meanwhile, OA imaging makes it possible to precisely control the timing of drug release. Furthermore, the in vivo thermal imaging provides more powerful auxiliary support for the application of PTX/si@MPDA-PEG in the treatment of ovarian cancer (Fig. 4F, S15). This not only helps to further confirm drug distribution in conjunction with OA imaging but also enables the evaluation of the treatment effect by monitoring the temperature changes. The mutual complementarity between in vivo thermal imaging and OA imaging provides abundant and reliable information for the early diagnosis of ovarian cancer and treatment monitoring.

Although PTX/si@MPDA-PEG has exhibited excellent anti-tumor efficacy, the specific mechanism between PTX and STMN1 remains elusive and warrants further in-depth exploration. Previous findings have indicated that stathmin promotes microtubule depolymerization by binding to α/β-tubulin heterodimers and forming a stable T2S complex, which accelerates GTP hydrolysis and prevents microtubule assembly [29, 44–46]. Meanwhile, the N-terminal hairpin structure of stathmin binds to α-tubulin, blocking the interaction between the T2S complex and microtubule protofilaments, and ultimately inhibiting microtubule assembly [45]. Furthermore, our results in Fig. 3G-H demonstrated that silencing STMN1 markedly increased acetylated α-tubulin, a marker of microtubule stabilization, indicating that reduced STMN1 expression directly enhances microtubule stability [27]. Consistently, previous studies have also shown that depletion of STMN1 induces extensive microtubule stabilization and alters cytoskeletal organization in various cell types, including hematopoietic progenitor cells [47] and cancer models [48, 49].

Based on the existing research findings and our results, we pr*opose* that a reduction in STMN1 expression leads to an increase in the concentration of free tubulin within the cell. This elevated level of free tubulin provides additional substrates for microtubule polymerization, thereby promoting the polymerization process. Simultaneously, the increased availability of free tubulin offers more binding sites for PTX, enabling PTX to more effectively inhibit microtubule depolymerization and enhance the sensitivity of ovarian cancer cells to PTX-based chemotherapy. This heightened sensitivity ultimately results in the arrest of the ovarian cancer cells during the G2/M phase of the cell cycle. Collectively, these mechanistic observations establish STMN1 as a key determinant of ovarian cancer cell responsiveness to PTX, while further validating our co-delivery system as a rational, targeted therapeutic strategy to surmount PTX chemoresistance in ovarian cancer—addressing a critical unmet need in clinical management of this disease.

## Conclusion

In this paper, we report a multifunctional nanoparticle that enables laser-controlled release of co-loaded paclitaxel (PTX) and STMN1 siRNA for synergistic combination therapy. This nanoparticle can improve the microtubule stability by interfering the expression of STMN1, thereby increasing the sensitivity to PTX, blocking cells in the G2/M phase, and ultimately leading to the death of ovarian cancer cells. Our nanoparticle offers an innovative strategy for the concurrent therapy and monitoring of ovarian cancer, enhancing therapeutic efficacy and diagnostic precision.

## Materials and methods

### Materials

Dopamine hydrochloride (98%), 1,3,5-trimethylbenzene (TME), ammonia solution (25–28%), Pluronic F127, and paclitaxel were purchased from Aladdin Reagent Co., Ltd. (Shanghai, China). The Calcein AM/PI double staining kit and cell cycle assay kit were purchased from Elabscience. The cell counting kit was purchased from Hangzhou Fude Biological Technology Co., Ltd. siRNA targeting human stathmin1 mRNA, including hSTMN1-291 (sense sequence: 5’-CUGGAGGAAAUUCAGAAGAAATT-3’, antisense 5’-UUUCUUCUGAAUUUCCUCCAGTT-3’), hSTMN1-381 (sense sequence: 5’-GAGCACGAGAAAGAAGUGCUUTT-3’, antisense 5’-AAGCACUUCUUUCUCGUGCUCTT-3’), hSTMN1-552 (sense sequence: 5’-CGGAAGAACAAAGAAUCCAAATT-3’, antisense 5’-UUUGGAUUCUUUGUUCUUCCGTT-3’) and scrambled siRNA (si-NC) were purchased from Sangon Biotech (Shanghai) Co., Ltd.

## Immunohistochemical analysis

Ovarian cancer tissue microarray (OVC0901, Shanghai Outdo Biotech Co., Ltd.) was purchased for STMN1 detection. The protocol was conducted as described in the previous paper [50]. The STMN1 antibody (ab52630) was purchased from Abcam (UK).

## SiRNA transfection

The protocol followed our previous paper [50, 51]. The siRNAs targeting STMN1 were designed and synthesized by Sangon Biotech (Shanghai, China). Transfections were performed using Lipofectamine™ 2000 (Invitrogen, USA) according to the manufacturer’s instructions. The efficiency of knockdown was evaluated through Western blot analysis. A2780 and SKOV3 cells that were transfected with negative siRNA were used as controls, and non-siRNA were used as blank, siRNA381 was chosen for further experiments.

### Preparation and characterization of NPs

MPDA nanomaterials were synthesized by a previously reported method [51] with modifications. In detail, 1 g of Pluronic F127, 0.5 g of dopamine, and 1 mL of TMB were added to a mixed solution of 50 mL of water and 50 mL of absolute ethanol, and the emulsion was formed by ultrasonication for 20 min, followed by stirring at 600 rpm at room temperature. Then, ammonia water was added to the mixed solution with stirring to adjust the pH to 10, and the reaction was maintained for 3 h under continuous stirring. The product was collected by centrifugation and washed with acetone and ethanol several times to obtain MPDA nanoparticles. Then the product was redispersed in water. MPDA nanoparticles were modified with amine-terminated PEG (mPEG-NH_2_, Mw = 10k). The characterization methods of the materials were carried out as described in previous studies [52]. The infrared absorption characteristics were characterized on a NICOLET iS 50 (Thermo Fisher Scientific, USA).

### Loading of PTX and SiRNA

Briefly, different w/w ratios (PTX: MPDA = 0.1:1, 0.2:1, 0.4:1, and 1:1) were dispersed in the solubilizer under stirring at room temperature. PTX-loaded MPDA-PEG nanoparticles were then collected by centrifugation and washed with deionized water three times to remove the surface adsorbed PTX. Free PTX in the supernatant was analyzed using a Lambda 750 UV-vis-NIR. The drug loading content (DLC) and encapsulation efficiency (EE) were calculated according to the following equations:

$$\begin{aligned} \text{DLC}\% = \frac{{\rm W}_{\rm PTX} \text{-} {\rm W}_{\rm Free\;PTX} }{ {\rm W}_{\rm PTX/si@MPDA\text{-} PEG} } \times 100\% \\ EE\% = \frac{{\rm W}_{\rm PTX} \text{-} {\rm W}_{\rm Free\;PTX} }{ {\rm W}_{\rm PTX} } \times 100\%\end{aligned}$$

For siRNA loading, siRNA was mixed with nanoparticles in 1 mL of DEPC water, where the weight-to-weight (w/w) ratios of siRNA to nanoparticles were 0.1:1, 0.2:1, 0.3:1, 0.4:1, 0.5:1, and 1:1. The pre-prepared agarose gel was placed into the electrophoresis tank and 1×TAE buffer was added until it completely covered the gel. Next, the siRNA-loaded nanoparticles were thoroughly mixed with the loading buffer, and then carefully loaded into the designated wells. Concurrently, the DNA marker was loaded into an adjacent well. Finally, the banding patterns were visualized and documented using a gel documentation system.

### Laser-triggered PTX release

PTX/si@MPDA-PEG nanoparticles were irradiated with an 808 nm laser (2 W/cm^2^) for 5 min and then after a 2-hour interval, repeated for 6 cycles. Centrifuged before each measurement and the supernatant was analyzed using Lambda 750 UV-vis-NIR.

### Photothermal effect evaluation

ddH_2_O and different concentrations PTX/si@MPDA-PEG solutions were irradiated with laser (808 nm, 2 W/cm^2^), and the temperature changes were recorded every 30 s. For the 200 µg/mL concentration of the nanomaterials, 5 cycles of laser irradiation experiments were conducted, during which the temperature was measured and recorded every minute. In addition, images were taken with an infrared thermal imager throughout the irradiation process.

### Cell biology experiments

For the cell cytotoxic assay, 3T3 cells were cultured in DMEM containing 10% (v/v) FBS and were plated in 96-well culture plates for 24-hour incubation. Subsequently, the cells are treated with different concentrations of MPDA-PEG nanoparticle dispersion and incubated for 24 h. Finally, the cell counting kit-8 assay was used to measure the viability of the cells.

Therapeutic efficacy was evaluated using Calcein AM/PI double staining and CCK-8 assays according to the manufacturers’ instructions. Specifically, A2780 cells were cultured with RPMI-1640 culture medium supplemented with 10% (v/v) FBS at 37 °C for 24 h. Subsequently, different nanoparticles (MPDA-PEG, PTX@MPDA-PEG and PTX/si@MPDA-PEG) were added, and the cells were incubated for an additional 24 h. For the MPDA-PEG + Laser, PTX@MPDA-PEG + Laser and PTX/si@MPDA-PEG + Laser groups, A2780 cells were exposed to nanoparticles for 4 h followed by laser irradiation (808 nm, 2 W/cm^2^) for 5 min. The therapeutic effect was then assessed after a further 24-hour incubation period.

The wound healing, Transwell, and confocal microscopy assays were performed according to our previous paper [50]. For cell-cycle analysis, cells were collected and fixed overnight in 70% ethanol at −20 °C, washed and resuspended in 100 µL RNase A Reagent for 30 min at 37 °C, then treated with 400 µL propidium iodide (PI) for 30 min at 4 °C in the dark. After staining, cell-cycle analyzes were carried out by flow cytometry. Data were analyzed with the FlowJo software.

### Western blotting

The experimental protocol was conducted as in our previous paper [53]. Briefly, protein extraction was performed using a commercial protein extraction buffer (PC201-1, Epizyme, China). For lysis, samples were incubated on ice for 5 min to ensure complete protein release, and protein quantification was conducted with the BCA quantification method (P0012, Beyotime, China). The STMN1 antibody (ab52630, 1:2000, Abcam, UK), the ace-a-tubulin antibody (5335 S, 1:2000, CST, China) and GAPDH (AB0037, 1:1000, Abways, China) were used for Western blot analysis.

### OA imaging in vitro and in vivo

Different concentrations of PTX/si@MPDA-PEG materials were performed under a 3D OA imaging system (LOIS-3D, TomoWave Suzhou Medical Imaging Co., Ltd., China).

For in vivo OA imaging (20 µL, 4 mg/mL) of PTX/si@MPDA-PEG, it was administered either intratumorally or via the tail vein of homozygous nude mice. Subsequently, the OA signals from the tumors at different time points after the injection were performed under the same 3D OA imaging system (LOIS-3D, TomoWave Suzhou Medical Imaging Co., Ltd, China).

### Evaluation of antitumor efficacy in vivo

For in vivo cancer therapy, tumor-bearing nude mice were randomly assigned to seven groups (*n* = 7): PBS (NC), PTX@MPDA-PEG (PMP), PTX/si@MPDA-PEG (PsMP), PBS + Laser (Laser), MPDA-PEG + Laser (MP + Laser), PTX@MPDA-PEG + Laser (PMP + Laser), PTX/si@MPDA-PEG + Laser (PsMP + Laser). When tumors reached a predetermined size, drugs were administered via intratumoral injection. For laser-treated groups, irradiation (808 nm, 2 W/cm²) was applied for 10 min at 4 h post-injection. The tumor volume and body weight of the mice were monitored every other day over a 14-day period.

On the third day, one nude mouse from each group was selected for sacrifice, and the tumors were harvested, fixed, and sectioned for hematoxylin-eosin (H&E), Ki-67, and TUNEL staining analysis. Ultimately, all remaining nude mice were sacrificed, and their tumor tissues were photographed. Subsequently, major organs from all groups were collected for histological examination via H&E staining, and blood samples were obtained for routine hematological and biochemical analyzes.

### Statistical analysis

The images were analyzed using ImageJ. All data analyzes were processed using GraphPad Prism 9.0 and Origin 2024. All data in this paper were expressed as mean ± SD. For statistical comparisons: Unpaired Student’s t-test was used for analyses involving two independent groups; For analyses involving three or more treatment groups, one-way analysis of variance (one-way ANOVA) was performed first, followed by Tukey’s post-hoc test to determine pairwise differences between groups. A probability (*p*) less than 0.05 was considered statistically significance.

## Supplementary Information

Supplementary Material 1.

## Data Availability

Data sharing is not applicable to this article as no new data were created or analyzed in this study.
