# Supplementary material for: Synergistic effect study on the co-delivery of paclitaxel and SiRNA targeting STMN1 based on MPDA nanoparticles in the therapy of ovarian cancer
Source: J Nanobiotechnology. 2025 Dec 9;23:763. doi: 10.1186/s12951-025-03827-8 (PMC12687539; doi:10.1186/s12951-025-03827-8)
Supplement: Supplementary file 1 — Supplementary Material 1. [file 12951_2025_3827_MOESM1_ESM.docx]

**Supplementary figures**

**
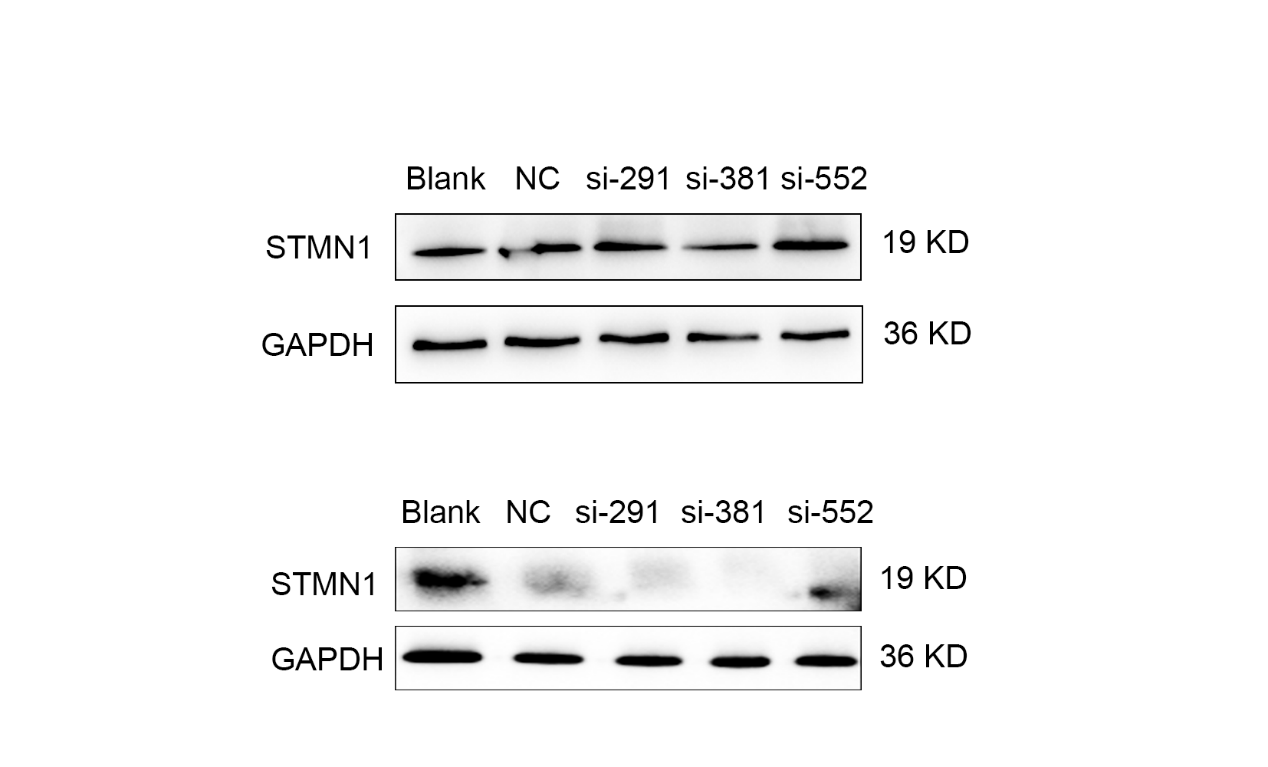
**

**Figure S1** Expression of STMN1 after transfection of three siRNAs in A2780 cells.


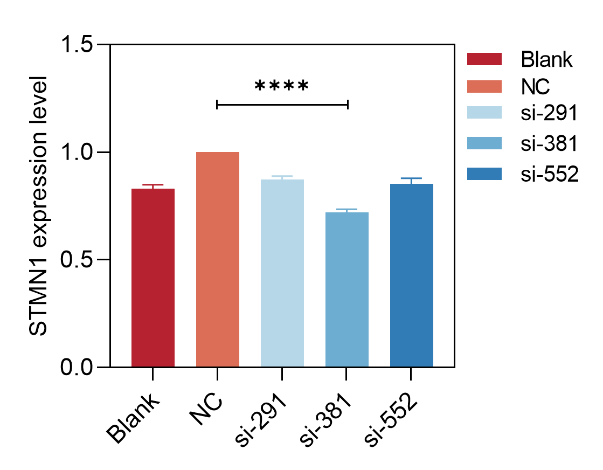


**Figure S2** Statistical analysis of STMN1 expression graph.

**
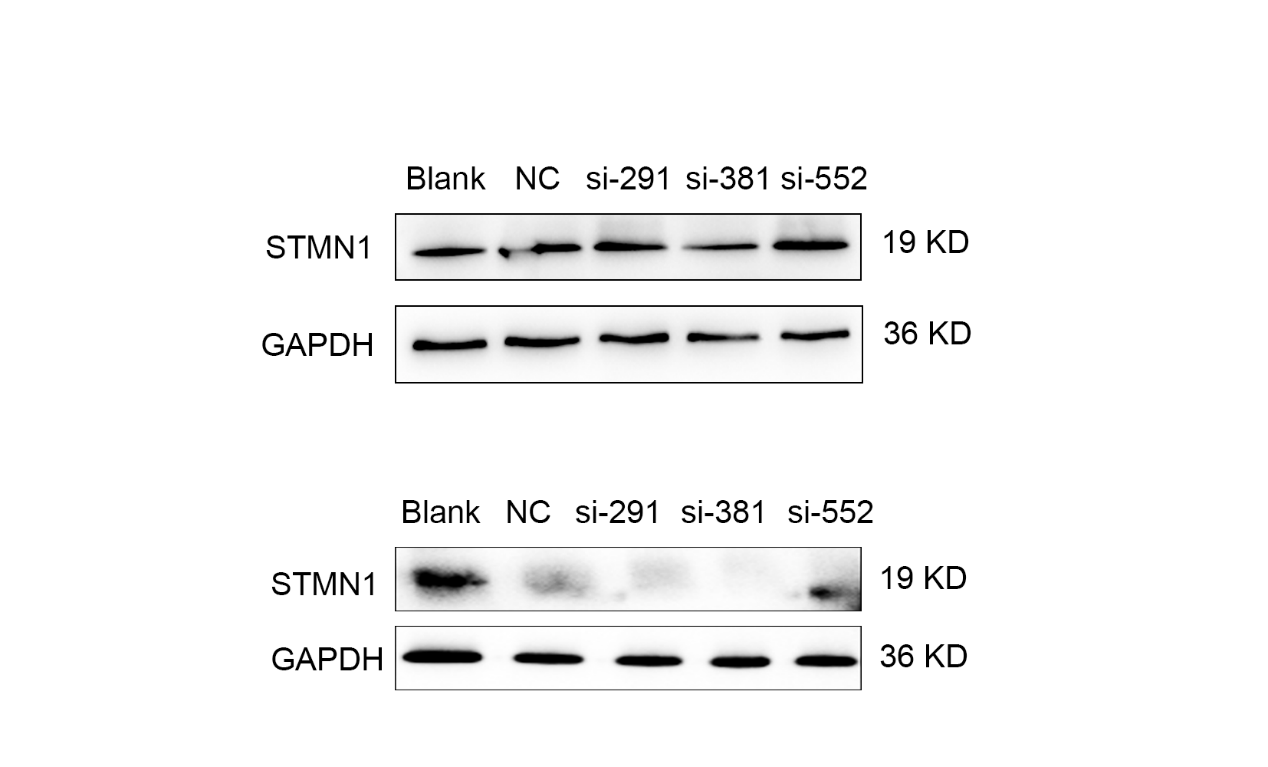
**

**Figure S3** Expression of STMN1 after transfection of three siRNAs in SKOV3 cells.


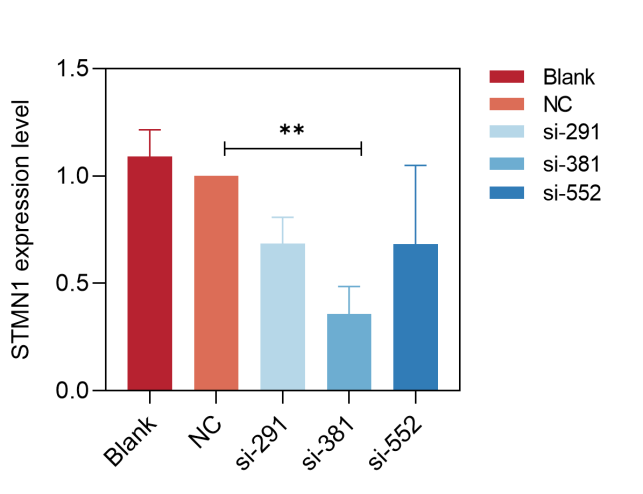


**Figure S4** Statistical analysis of STMN1 expression graph.


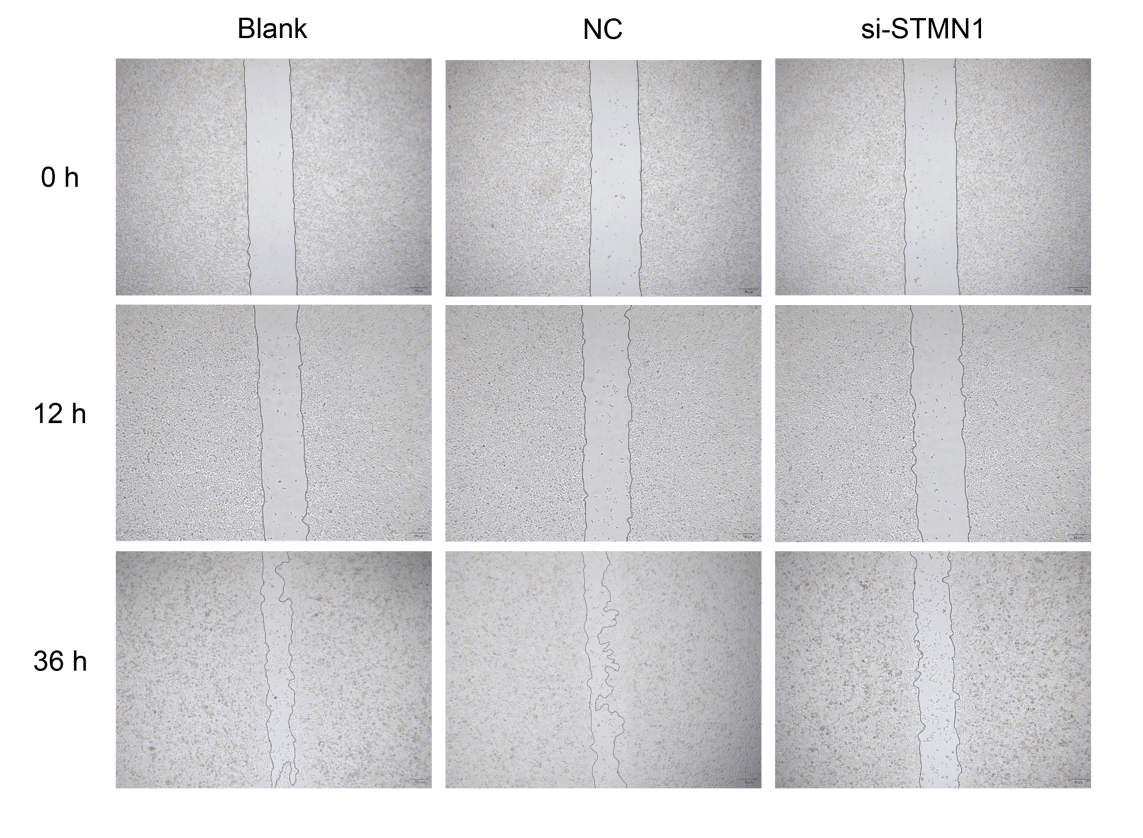


**Figure S5** Representative images of the healing of A2780 cellular scratches with different treatments for 0, 12, and 36 h.

**
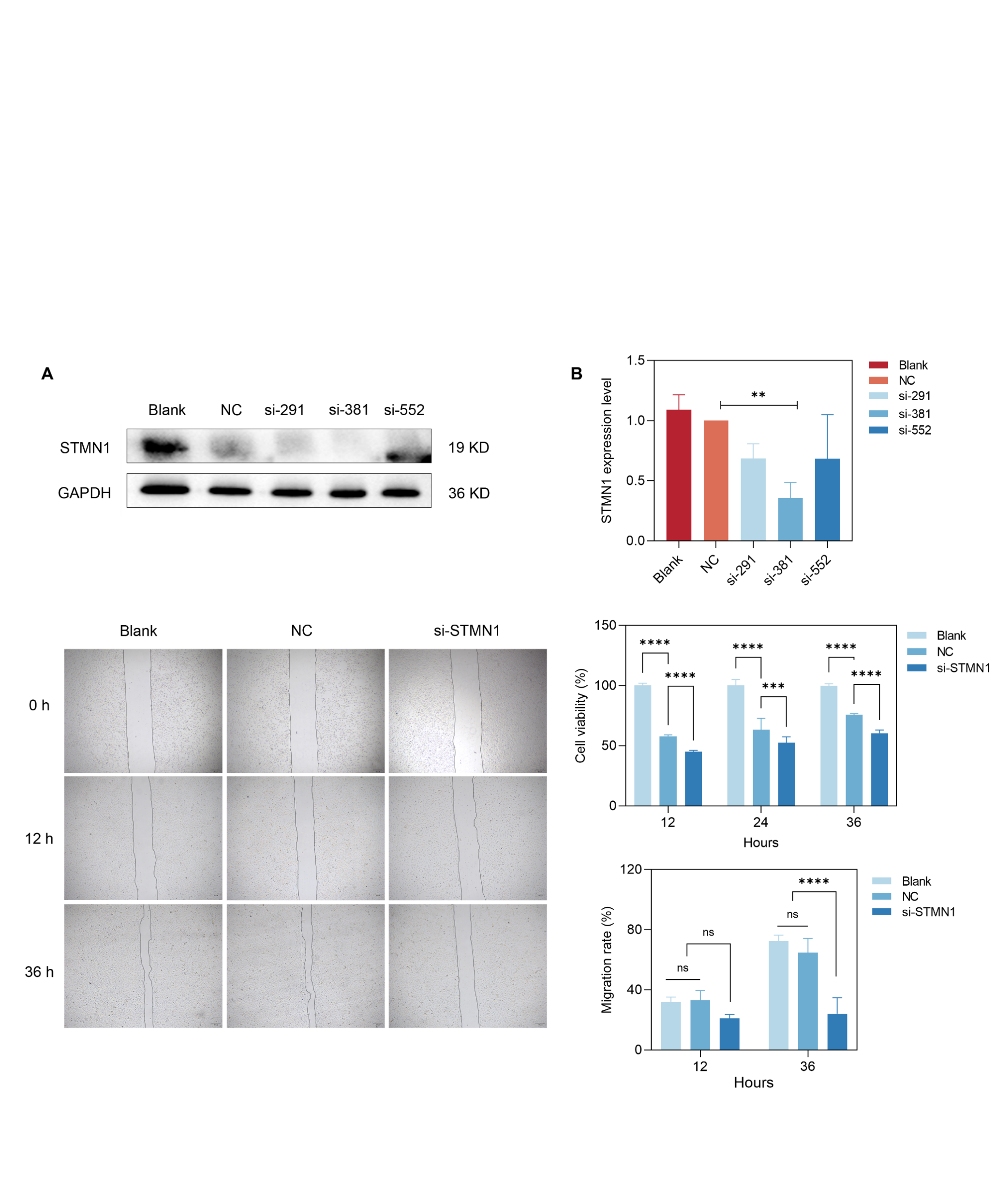
**

**Figure S6** Representative images of the healing of SKOV3 cellular scratches with different treatments for 0, 12, and 36 h.


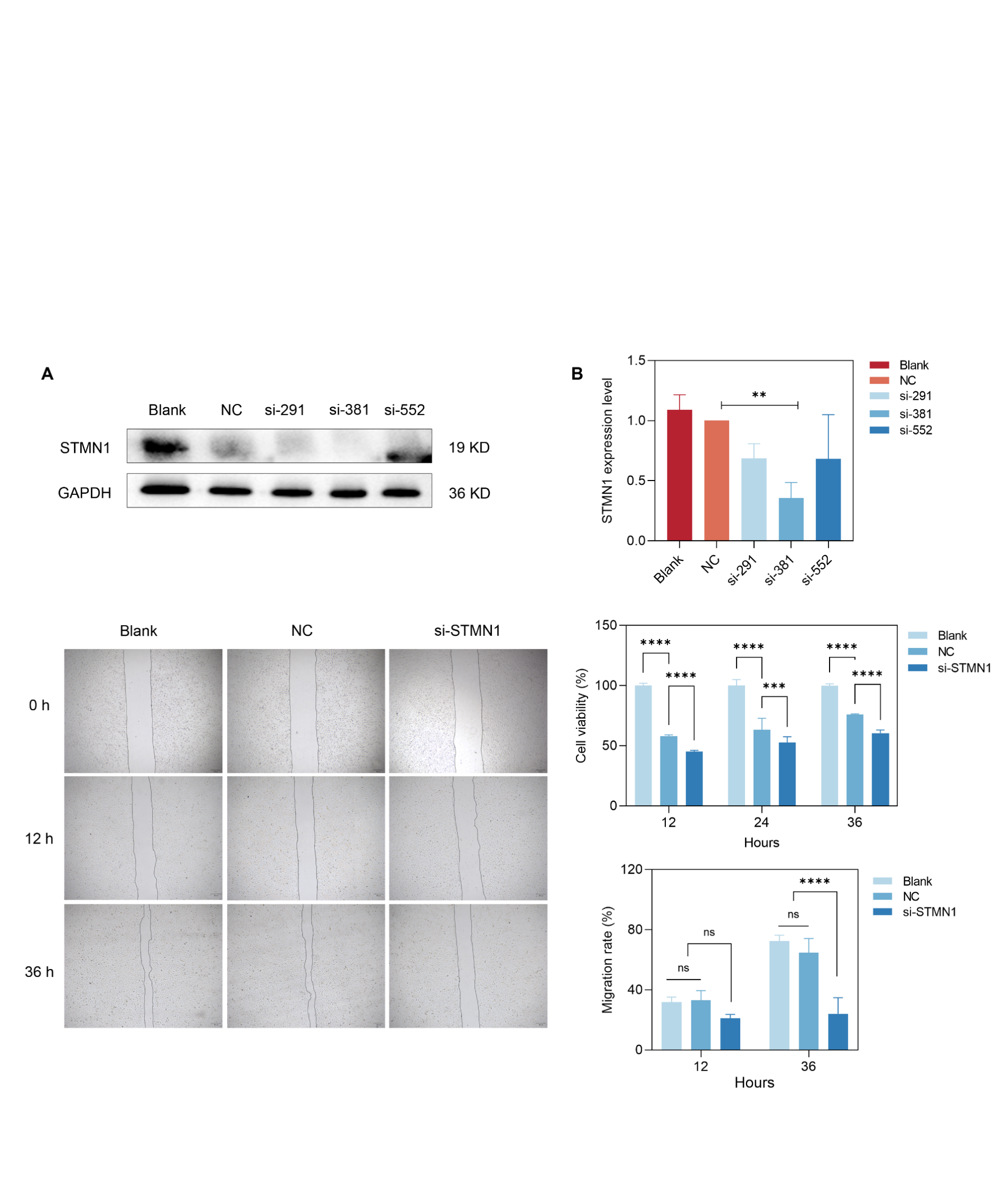


**Figure S7** Analysis of wound healing in the wound healing assay in different groups. **
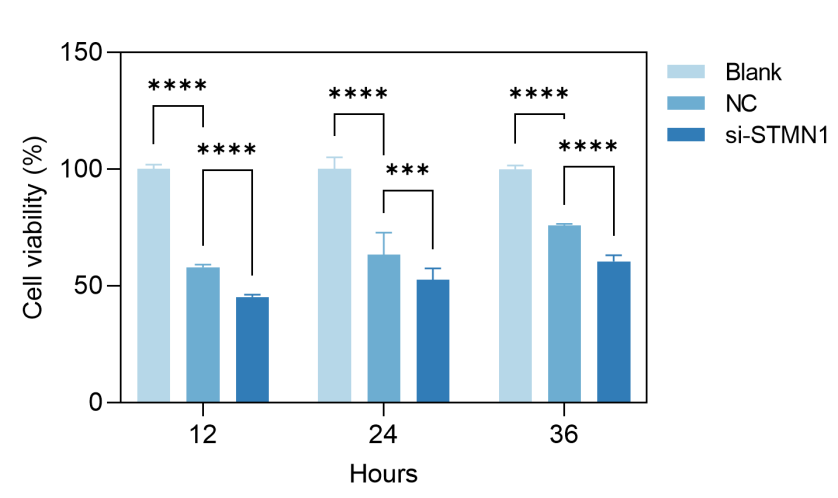
**

**Figure S8** Cell viability in different treatment groups at 24 and 48 hour, as measured by the CCK-8 assay.


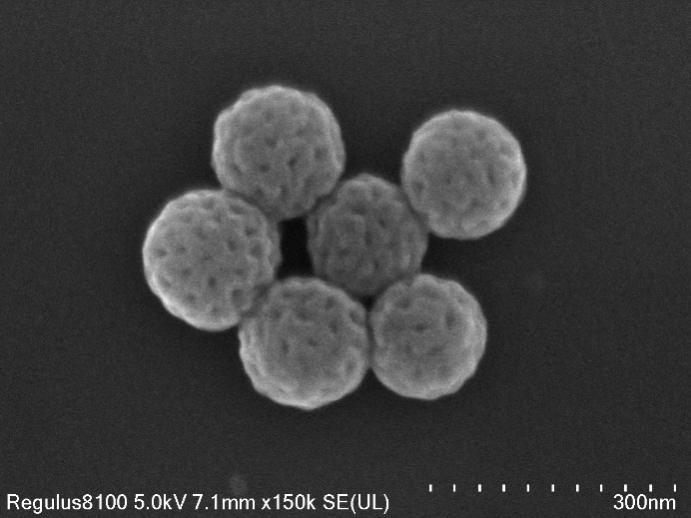


**Figure S9** Scanning electron microscope image of mesoporous polydopamine nanoparticles (~150 nm).


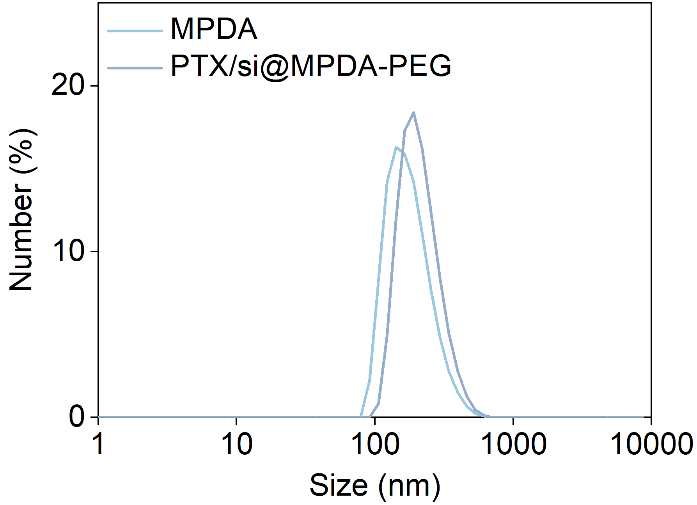


**Figure S10** DLS measurements of MPDA and PTX/si@MPDA-PEG


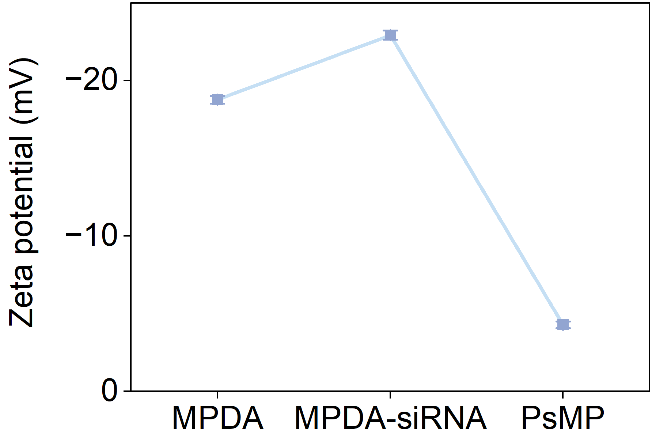


**Figure S11** Zeta potential of MPDA and PTX/si@MPDA-PEG


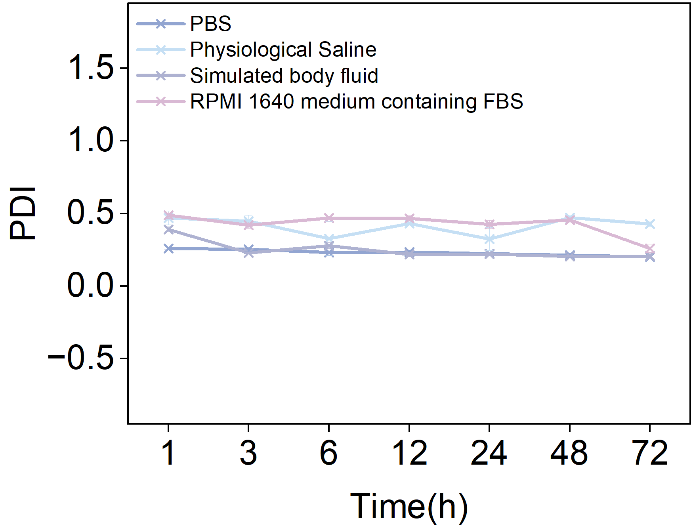


**Figure S12**  Polydispersity index (PDI) versus time (1-72 h) in different solution environments.


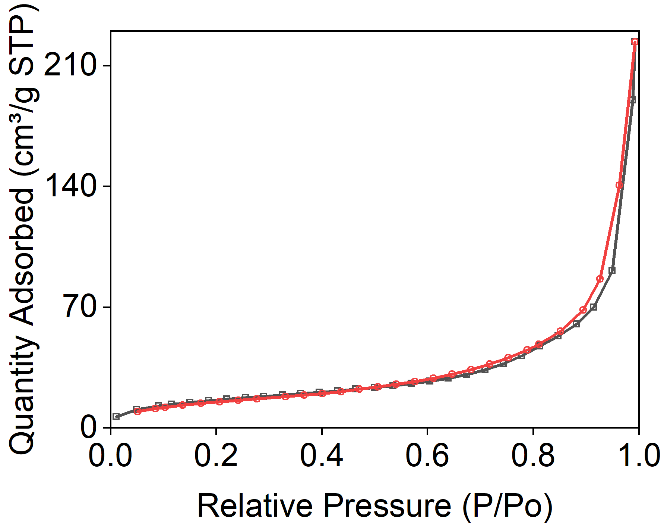


**Figure S13** Nitrogen adsorption-desorption isotherms


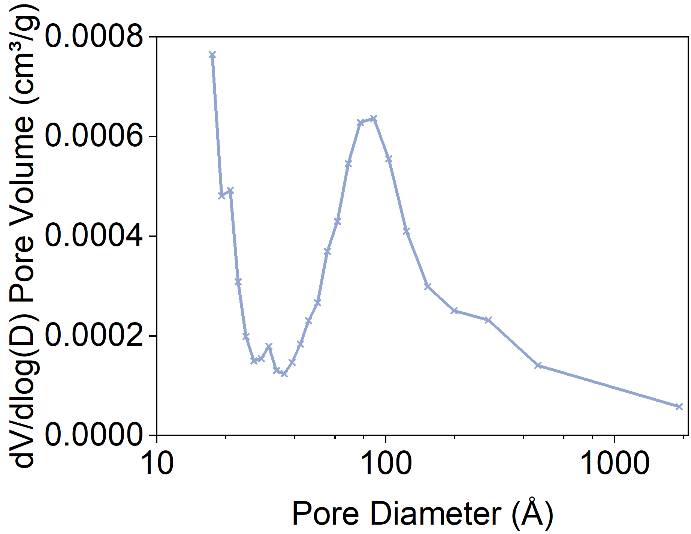


**Figure S14** Pore size distribution curves of MPDA


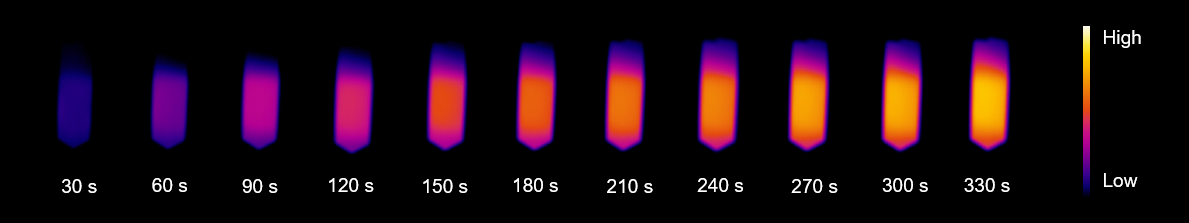


**Figure S15** Thermal infrared images of PTX/si@MPDA-PEG solutions exposed to 808 nm laser irradiation for 300 seconds.

**
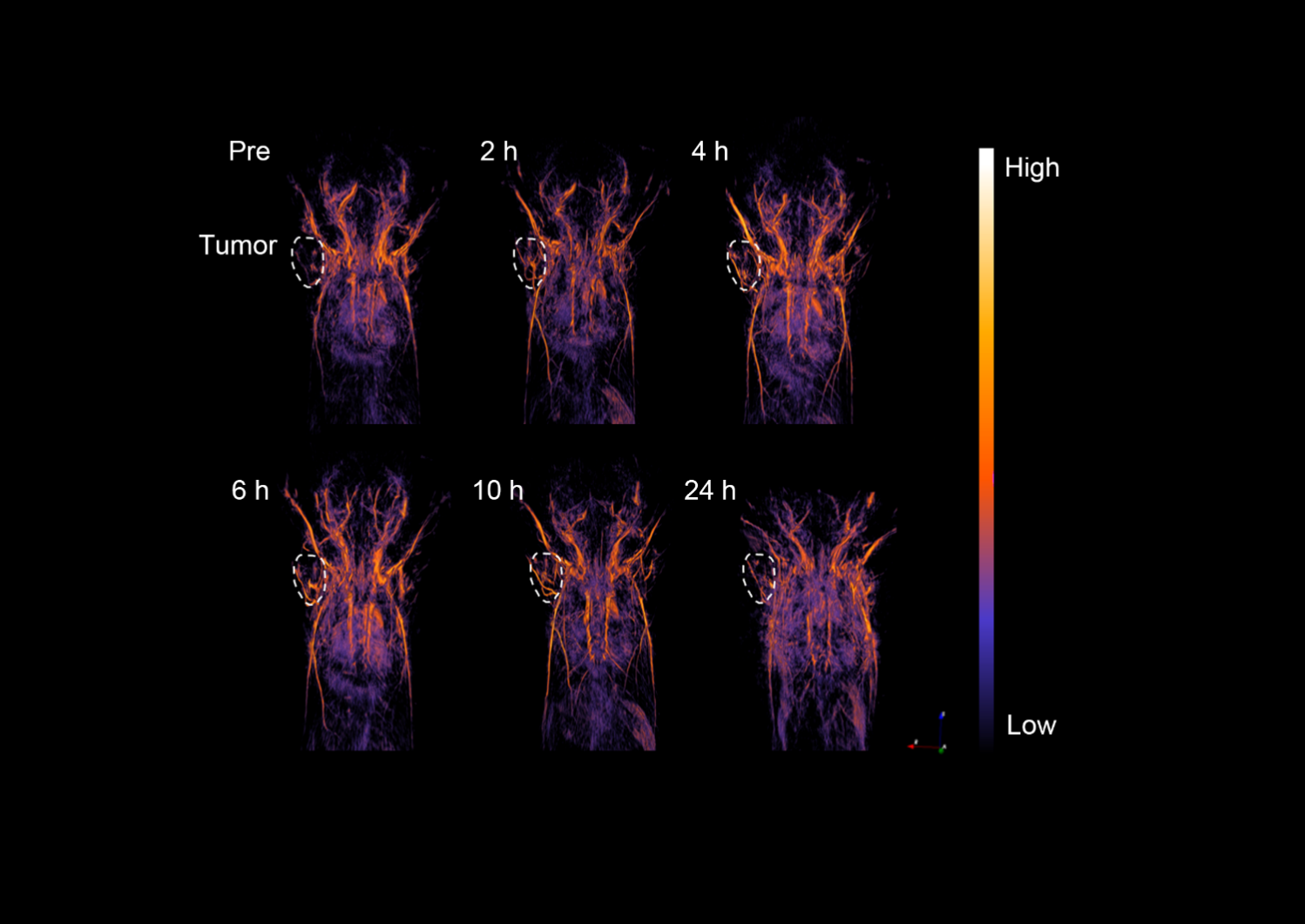
**

**Figure S16** Time-dependent PA images of tumor sites after tail vein injection of PTX/si@MPDA-PEG nanoparticles in tumor-bearing nude mice.


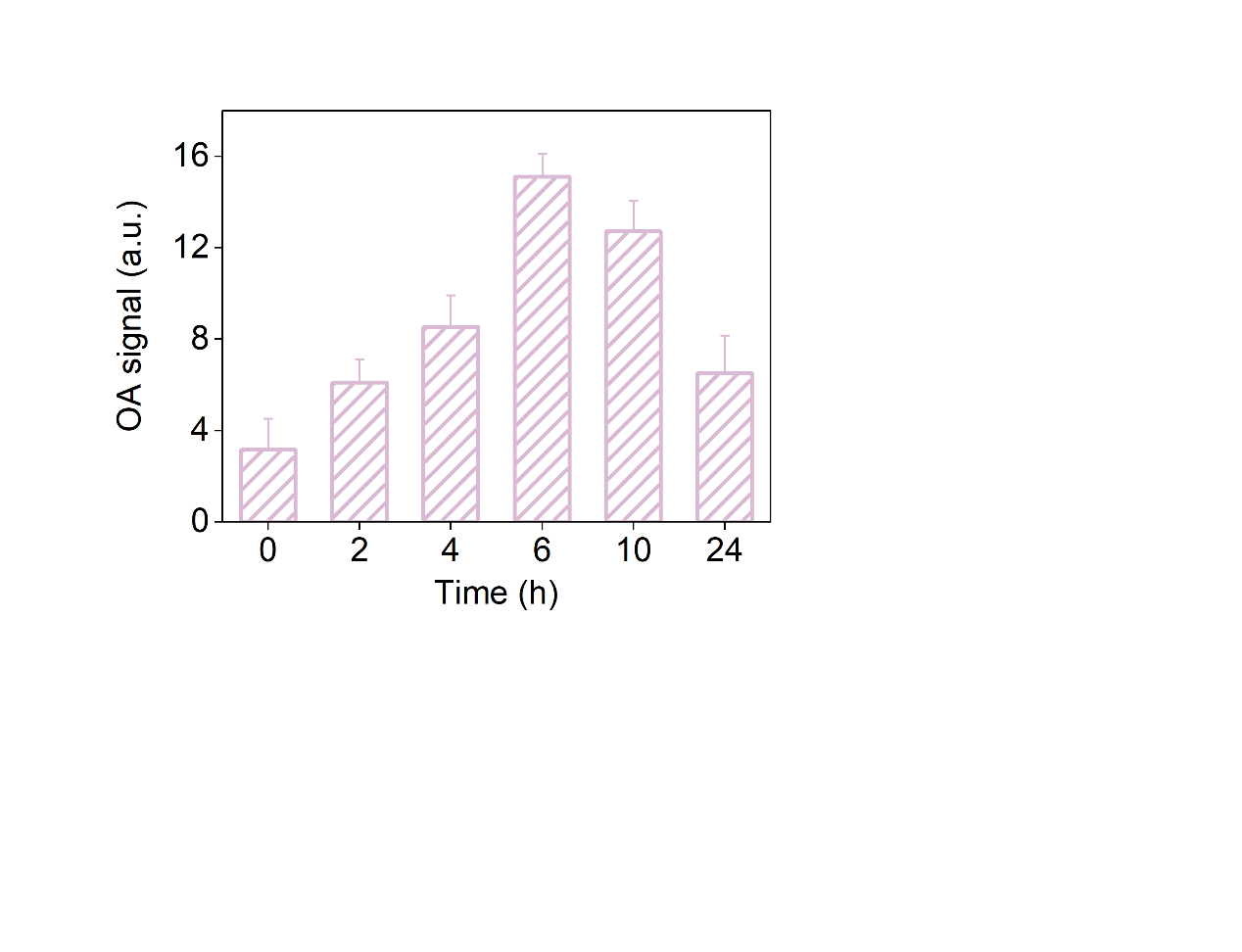


**Figure S17** OA signal in the tumor region after injected PTX/si@MPDA-PEG nanoparticles are recorded at different time points.


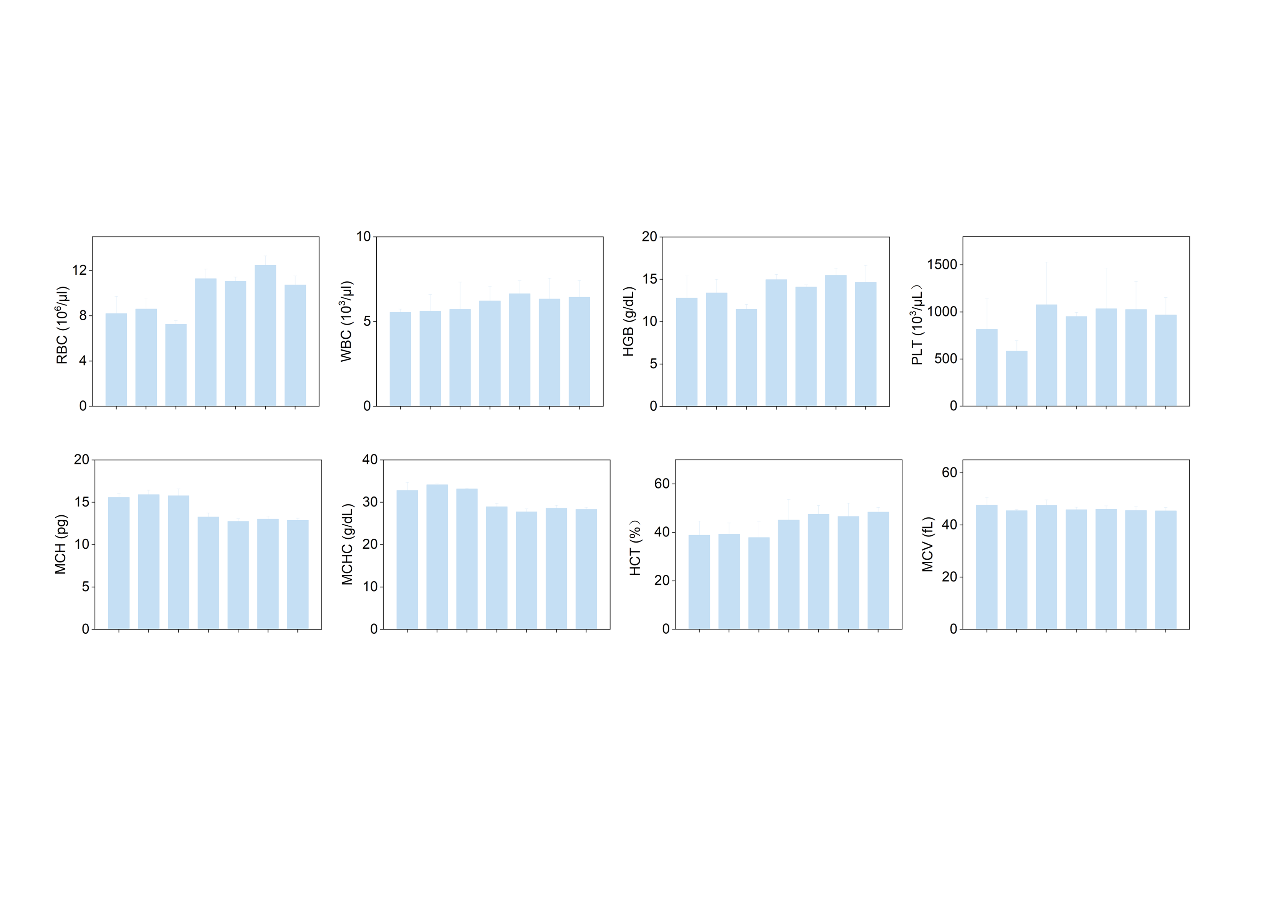


**Figure S18** The hematology analysis of nude mice in different groups after 14 days.
